# Supplementary material for: A non-invasive method to genotype cephalopod sex by quantitative PCR
Source: iScience. 2026 Apr 3;29(5):115594. doi: 10.1016/j.isci.2026.115594 (PMC13122204; doi:10.1016/j.isci.2026.115594)
Supplement: Document S1. Figures S1–S5, Table S1 [file mmc1.pdf]

iScience, Volume *29*

Supplemental information

A non-invasive method to genotype cephalopod sex by quantitative PCR

Frederick A. Rubino, Gabrielle C. Coffing, Connor J. Gibbons, Scott T. Small, Thomas Desvignes, Jeffrey Pessutti, Ann M. Petersen, Alexander Arkhipkin, Zhanna Shcherbich, John H. Postlethwait, Andrew D. Kern, and Tessa G. Montague

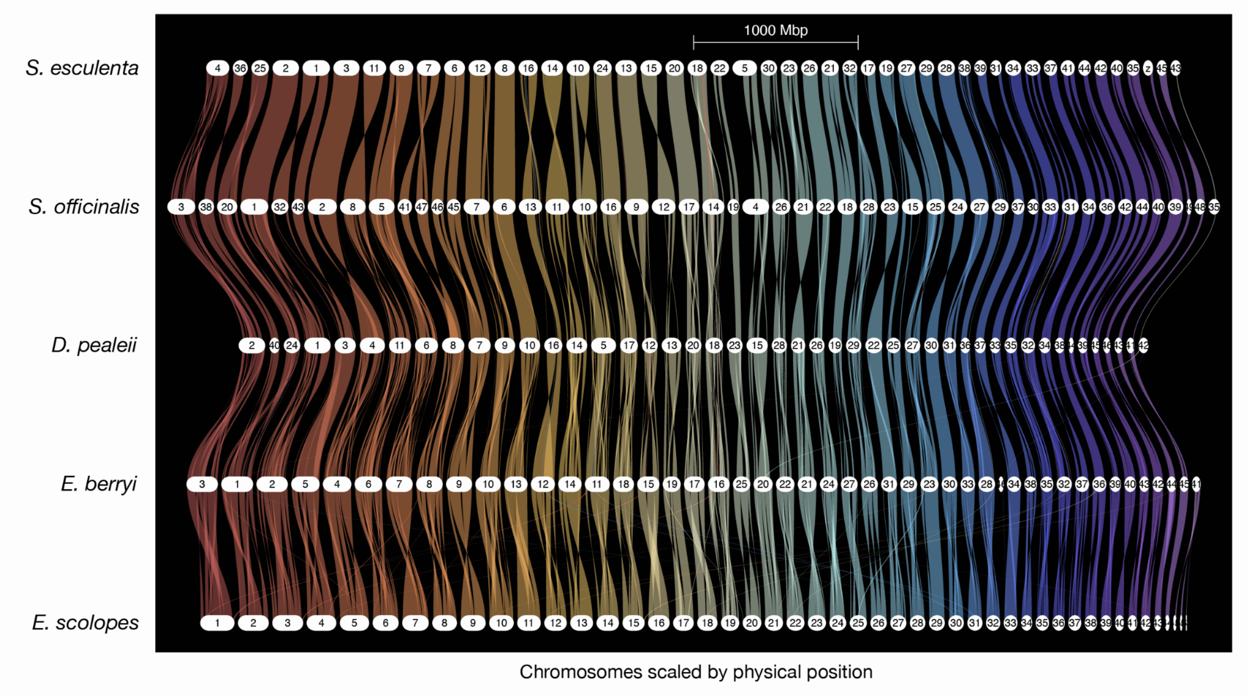

Figure S1. Conserved synteny relationships among chromosomes of *E. scolopes, E. berryi, D. pealeii, S. officinalis*, and *S. esculenta,* Related to Figure 2. This riparian synteny plot was generated from orthogroups with *E. scolopes* set as the reference species.

Organism	Assembly ID	Reference	Primer name	Primer sequence (5'→3')	Chromosome/scaffold name	Amplicon coordinates	Gene ID	Amplicon size (bp)	Efficiency (range)	Confirmation of specificity	Amplicon sequence
Ascarosepion bandense	GCA_037127315.1	Lorig-Roach et al. 2024	Abandense_qPCR_auto_F<br>Abandense_qPCR_auto_R	GGTGTTGTTCGCTCAGTTATC<br>GTGTTCATGTCGCCATCTTATC	JBBBDQ010000781.1	JBBBDQ010000781.1:3915044-3914906	MSTRG.6212	139	110% (Cq ~ 23-28)<br>4 - 0.1 ng/μL	- Single band of expected size by gel electrophoresis<br><br>- Single melt curve following qPCR<br><br>- Single product of expected sequence by nanopore sequencing<br><br>(see Figure S2)	GGTGTTGTTCGCTCAGTTATCTGGACTTTTCCGCTTATCTGAAAGCAAGGCCAGTGCCCTTTAGCCTGGACCCAATAATCTTGGCAACCCCT<br>TATTCTGTTAAGAATGTTGAAGTTAGATAAGATGGCGACATGAACAC
			Abandense_qPCR_sex_F<br>Abandense_qPCR_sex_R	GGCCGTCCTCTACTTGTAATG<br>AGTAGCTGTGTGGTTGAGAAG	JBBBDQ010002443.1	JBBBDQ010002443.1:221249-221378	MSTRG.16042	130	107% (Cq ~ 23-28)<br>4 - 0.1 ng/μL		GGCCGTCCTCTACTTGTAATGATGATAGTGGTGGTGACGCTAACAGAGAGGGGAGAAGATTCATACGTTTCACGTTACACCTCTCTCTCTCT<br>ATTCAATTTTACTTTATCTTCTCAACCACACAGCTACT
Octopus bimaculoides	GCA_001194135.2	Albertin et al. 2022	Obimaculoides_qPCR_auto_F<br>Obimaculoides_qPCR_auto_R	TTGTTTGGACCTTGGGCTTATAG<br>CTGTCATGAACCCTGGTGTATTC	1	1:21853962-21854061	LOC106878076	100	107% (Cq ~ 23-28)<br>3 - 0.1 ng/μL		TTGTTTGGACCTTGGGCTTATAGAAACAATCATGGTTTTTGGGCAATAGATGCTGCAGGGTTGAAAGAGAGTTATTTGAATACACCAGGGTT<br>CATGACAG
			Obimaculoides_qPCR_sex_F<br>Obimaculoides_qPCR_sex_R	CCTCACCACTGGATGCAATTAAG<br>GCCAATCCGTCCAACCTATAC	17 (Z)	16:55571227-55571384	LOC106875932	158	110% (Cq ~ 23-30)<br>5 - 0.04 ng/μL		CCTCACCACTGGATGCAATTAAGAGTTATACACAAGACATGCAGTAACAACTGGTAGCAGCTGCAGTTGTAGTGGTGATAGTGAGAGCAGCA<br>ATCATCATCATCATCATCATTATTTAACACCCATGTTCTATGCTGGTATAGGTTGGACGGATTGGC
Sepia officinalis	GCA_964300435.1	Darwin Tree of Life Project Consortium, 2022	Sofficinalis_qPCR_auto_F<br>Sofficinalis_qPCR_auto_R	TTTGCCACTGTGTCCCTTTATAC<br>ACACACACAGGCTGCTTATTG	1	1:63618568-63618747	SPHA_17994	180	106% (Cq ~ 22-29)<br>10 - 0.1 ng/μL		TTTGCCACTGTGTCCCTTTATACCTTCTCTTTCACCTGTAAGCGAACTGCGTTGGGCGTCACGTGGACAATAGTGACTCCGATGGGTAACGA<br>GAAGGTGTGTTGAAGGGTGATGCTGAGAGGCGTCTGTTTGGGGTTCACTGACTCGAAATTGGCCGGACAATAAGCAGCCTGTGTGTGT
			Sofficinalis_qPCR_sex_F<br>Sofficinalis_qPCR_sex_R	TTTCAACCCATCTGCGTCTATAG<br>ACTCCTCTCGTTGCATGATTAC	49 (Z)	49:22163919-22164086	SPHA_41527	168	108% (Cq ~ 23-28)<br>12 - 0.4 ng/μL		TTTCAACCCATCTGCGTCTATAGATGCGCCTGCGCGCACACACACATGTTGCTAATTTACCAGTTAGCTGTAGAGATGTACGCTTAAAAC<br>TGATAGATATGTAGGTAGGTAGATAGTTATGCAAGTAGATAGGTTGGTATGTGGGTAATCATGCAACGAGAGGAGT
Euprymna berryi	Unpublished assembly: https://drive.google.com/drive/folders/1Cf8pB8_S0YmZF3i3l2ZfA3jpDhsDaoYR	Gavriouchkina et al. 2025	Eberryi_qPCR_auto_F<br>Eberryi_qPCR_auto_R	CTTTCGCCACGCCTGATATAC<br>CAGCAGCTTCTTTCCCAGATAAG	2	2:152813844-152813707	EB27798	138	90% (Cq ~ 21-30)<br>30 - 0.06 ng/μL		CTTTCGCCACGCCTGATATACATTTTTGTTTATTATAGTGCATCATAATCAACTAGTGGAATGAAACCTTTCCTGTTCACAATTATTCTTAT<br>AGTTGATAGCATCGAGTTTACATCTTATCTGGGAAAGAAGCTGCTG
			Eberryi_qPCR_sex_F<br>Eberryi_qPCR_sex_R	CTGCCCAGCGAATTGTTTATTG<br>TCCGGCGTCTAGGGATTTAG	44 (Z)	44:27582972-27582786	EB34814	187	99% (Cq ~ 24-28)<br>7 - 0.5 ng/μL		CTGCCCAGCGAATTGTTTATTGGGGCTGTTGCACACGTTTGTACCGACTATAACGGTTGGTTTGGCGAGACTCGTATCAAAGCTCAAGTTAC<br>CAAGGGACTGATCACATTACTGAAGAAACAGGGCATGGTATAGCGGAAAGGAACGAGACGAAACGATCACAGTTTCTAAATCCCTAGACGCC<br>GGA
Doryteuthis pealeii	GCA_023376005.1	Albertin et al. 2022	Dpealeii_qPCR_auto_F<br>Dpealeii_qPCR_auto_R	CACTTCAGCCCGATGGAATAAG<br>CTTTGTAAATGCCGCACCTATATC	1	1:148199925-148200024	Dopeav2001646m.g	100	101% (Cq ~ 23-31)<br>4 - 0.02 ng/μL		CTTTGTAAATGCCGCACCTATATCGATGTATGTAGTATGGTCACCTCATTGACCGTATTATTCGTATCGTTGTTTCCACTTATTCCATCGGG<br>CTGAAGTG
			Dpealeii_qPCR_sex_F<br>Dpealeii_qPCR_sex_R	GGAGTCTGAGGTCCGAGATATAG<br>GCCGAGACCACAAACAATAAC	43 (Z)	43:44041819-44041958	Dopeav2130852m.g	140	100% (Cq ~ 22-31)<br>12 - 0.02 ng/μL		GCCGAGACCACAAACAATAACGCATTTCATGGTCCTAGCAAAACTTACACTTCTCAGTCTCGTCCGGATCCGCCGGATCCTGATCCGGACCG<br>ACCTTAGGAAACATATTATCAAATTCTATATCTCGGACCTCAGACTCC
Illex illecebrosus & Illex argentinus	Low-coverage whole-genome sequencing (lcWGS) Illumina library	This study	Illex_qPCR_auto_F<br>Illex_qPCR_auto_R	AAAACTCCCGACGTCTTGAA<br>GGCCATCCTGGTAGACAAGA	N/A	homology to *D. pealeii*<br>1:65236100-65236256	N/A	160	98% (Cq ~ 21-28)<br>10 - 0.08 ng/μL		AAAACTCCCGACGTCTTGAAGGACAAATTGAGGGAAGCAATGAGAACAGGAGACAACGACGCTCTGGAAGCTGCTCTTGACGAGTGTGTTGC<br>CGCTGGCATGCCAGAACTGGACGCCGATATTCACAGAGCAAGAAAAGCTCTTGTCTACCAGGATGGCC
			Illex_qPCR_sex_F<br>Illex_qPCR_sex_R	AATCACCCCAACCAGATGAA<br>CTCCTGGACCTGGAATGAAA	N/A	homology to *D. pealeii*<br>43:25792260-25792529	N/A	273	97% (Cq ~ 21-30)<br>11 - 0.02 ng/μL		AATCACCCCAACCAGATGAATCTGAATTAGCAGCTGCAGCAGACTTAGATGCTTCGGTTGTGTTTTTTGATGCTATTTCAGATGATGTTAAA<br>GGATTGTTCTTGCTGCCCGGAGCAGCTCCAATGGCAGATGTGTCTGCAGAAGAGGAATTTAGATCACCAAGAATATCCGACAAAGATGCAGA<br>TGAAGATGCGGCTGGGTTTGCTGAGGGCTGGCTCCCAACAGTTGGGCTAGATCCAGGAGGGGCAAGTATTTTCATTCCAGGTCCAGGAG

Table S1. Primer locus information, Related to Table 1.

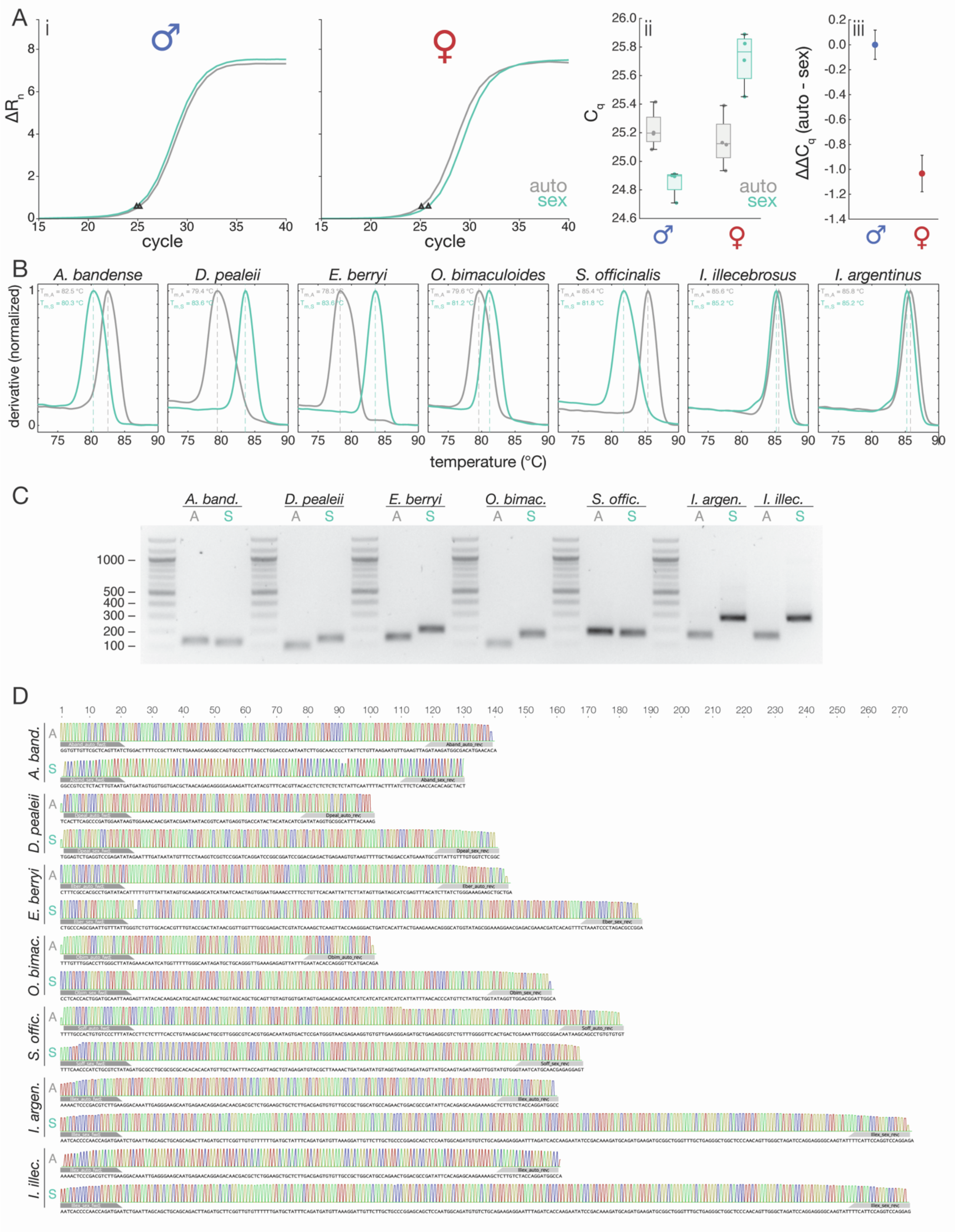

Figure S2. Validation of primer pairs used for quantitative PCR against autosome and sex chromosome loci of seven cephalopod species, Related to Figure 3. (A) Determination of $\Delta\Delta C_q$ values for a single male

and a single female standard of *A. bandense*: (i) Amplification curves of male and female standards using autosome- and sex chromosome-targeting primer pairs (specified in Table S1). ΔR_n is the change in normalized reporter fluorescence relative to the baseline signal. Triangles (△) represent C_q values as determined by QuantStudio Design & Analysis Software. For visual clarity, only a single technical replicate is shown for each; (ii) C_q values from four technical replicates of each reaction. Box plots show the interquartile range with median line; whiskers indicate minimum and maximum values. (iii) Normalized $\Delta\Delta C_q$ values determined from four technical replicates (see Quantitative PCR analysis in Methods). Error bars indicate the summation of standard error of the mean values of $C_{q,auto}$ and $C_{q,sex}$. (B) Melt curve analysis of all primer pairs used in this study (Table S1). Melting temperature (T_m) as determined by QuantStudio Design & Analysis Software is indicated by a dashed line. (C) Agarose gel electrophoresis of qPCR amplicons. (D) Raw signal trace from nanopore sequencing of qPCR amplicons, with primer binding sites indicated.

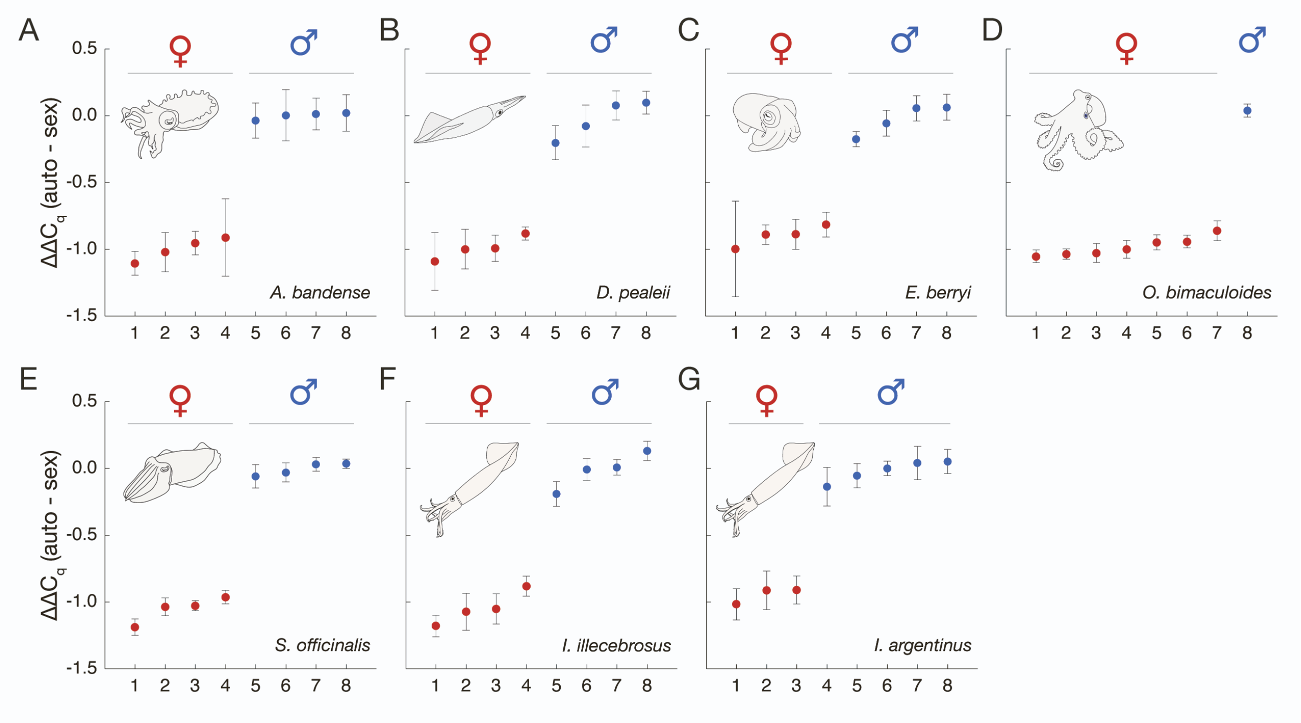

Figure S3. Quantitative PCR shows sex-specific dosage differences in seven octopus, cuttlefish, and squid species, Related to Figure 3. (A) *A. bandense.* (B) *D. pealeii.* (C) *E. berryi.* (D) *O. bimaculoides.* (E) *S. officinalis.* (F) *I. illecebrosus.* (G) *I. argentinus.* For each species, $\Delta\Delta C_q$ values were calculated between autosomal and sex-linked loci across eight blinded, randomized tissue samples, normalized such that the median value of the greater-$\Delta\Delta C_q$ cluster (male) was set to zero. Individuals are arranged along the x-axis in order of increasing $\Delta\Delta C_q$. Data points and error bars represent the mean $\Delta\Delta C_q$ value and standard error of the mean, respectively, of four technical replicates for a single animal (N = 8 animals for each species).

A

Autosome

```
                Illex.1:    1-  100
I. illecebrosus AAAACTCCCGACGTCTTGAAGGACAAATTGAGGGAAGCAATGAGAACAGGAGACAACGACGCTCTGGAAGCTGCTCTTGACGAGTGTGTTGCCGCTGGCA
                ||||||||||||||||||||||||||| |||||||||| ||||||| ||||||||| ||||||||||||| || |||||| || ||| | ||||||||||
D. pealeii      AAAACTCCCGACGTCTTGAAGGACAAACTGAGGGAAGCCATGAGAAGAGGAGACAAGGACGCTCTGGAAGATGTTCTTGATGAATGTATCGCCGCTGGCA
                Dpe01:65236255-65236100:    1-  100

                Illex.1:  101-  160
I. illecebrosus TGCCAGAACTGGACGCCGATATTCACAGAGCAAGAAAAGCTCTTGTCTACCAGGATGGCC
                |||||||| | ||||| |||||||||||||||||||||||||||| | ||||||||||||
D. pealeii      TGCCAGAATTAGACGCTGATATTCACAGAGCAAGAAAAGCTCTTGACGACCAGGATGGCC
                Dpe01:65236255-65236100:  101-  160
```

B

Sex chromosome

```
                Illex.Z:1-100
I. illecebrosus AATCACCCCAACCAGATGAATCTGAATTAGCAGCTGCAGCAGACTTAGATGCTTCGGTTGTGTTTTTTGATGCTATTTCAGATGATGTTAAAGGATTGTT
                |||| ||||| |||||||||||||| ||||||||||||||||||||||||||||| |||||||| |||||  ||||| ||||||||  || |||||||||
D. pealeii      AATCCCCCCAGCCAGATGAATCTGAGTTAGCAGCTGCAGCAGACTTAGATGCTTCAGTTGTGTTATTTGAATCTATTGCAGATGATAGTATAGGATTGTT
                Dpe43:25792259-25792529:1-100

                Illex.Z:101-200
I. illecebrosus CTTGCTGCCCGGAGCAGCTCCAATGGCAGATGTGTCTGCAGAAGAGGAATTTAGATCACCAAGAATATCCGACAAAGATGCAGATGAAGATGCGGCTGGG
                ||| ||||| |||||||||||||||||||||||||| |||| |  || ||| |  || ||||  | |||   |||||| ||||||||||||||||||||
D. pealeii      CTTACTGCCTGGAGCAGCTCCAATGGCAGATGTGTCCGCAG-A-TGG-ATTCATCTCGCCAAACAAATCTACCAAAGAGGCAGATGAAGATGCGGCTGGA
                Dpe43:25792259-25792529:101-197

                Illex.Z:201-273
I. illecebrosus TTTGCTGAGGGCTGGCTCCCAACAGTTGGGCTAGATCCAGGAGGGGCAAGTATTTTCATTCCAGGTCCAGGAG
                ||||||||| ||||| |||||||||||||||||||||||||||| ||||||||||||||| ||||||||||||
D. pealeii      TTTGCTGAGTGCTGGATCCCAACAGTTGGGCTAGATCCAGGAGGTGCAAGTATTTTCATTGCAGGTCCAGGAG
                Dpe43:25792259-25792529:198-270
```

Figure S4. Amplicons used for sex genotyping in *I. illecebrosus*, showing the alignment of *I. illecebrosus* reads to the *D. pealeii* genome, Related to Figure 3. (A) Autosomal amplicon (aligned to *D. pealeii* chromosome 1). Red line marks the *I. illecebrosus* primers. The forward primer has no mismatches, while the reverse primer has mismatches at position 13 (C>A) and position 15 (T>A). (B) Sex (Z) chromosome amplicon (aligned to *D. pealeii* chromosome 43). Blue line marks the *I. illecebrosus* primers. The forward primer has two mismatches (position 5 C>A and position 11 G>A), while the reverse primer has one mismatch at position 13 (C>G).

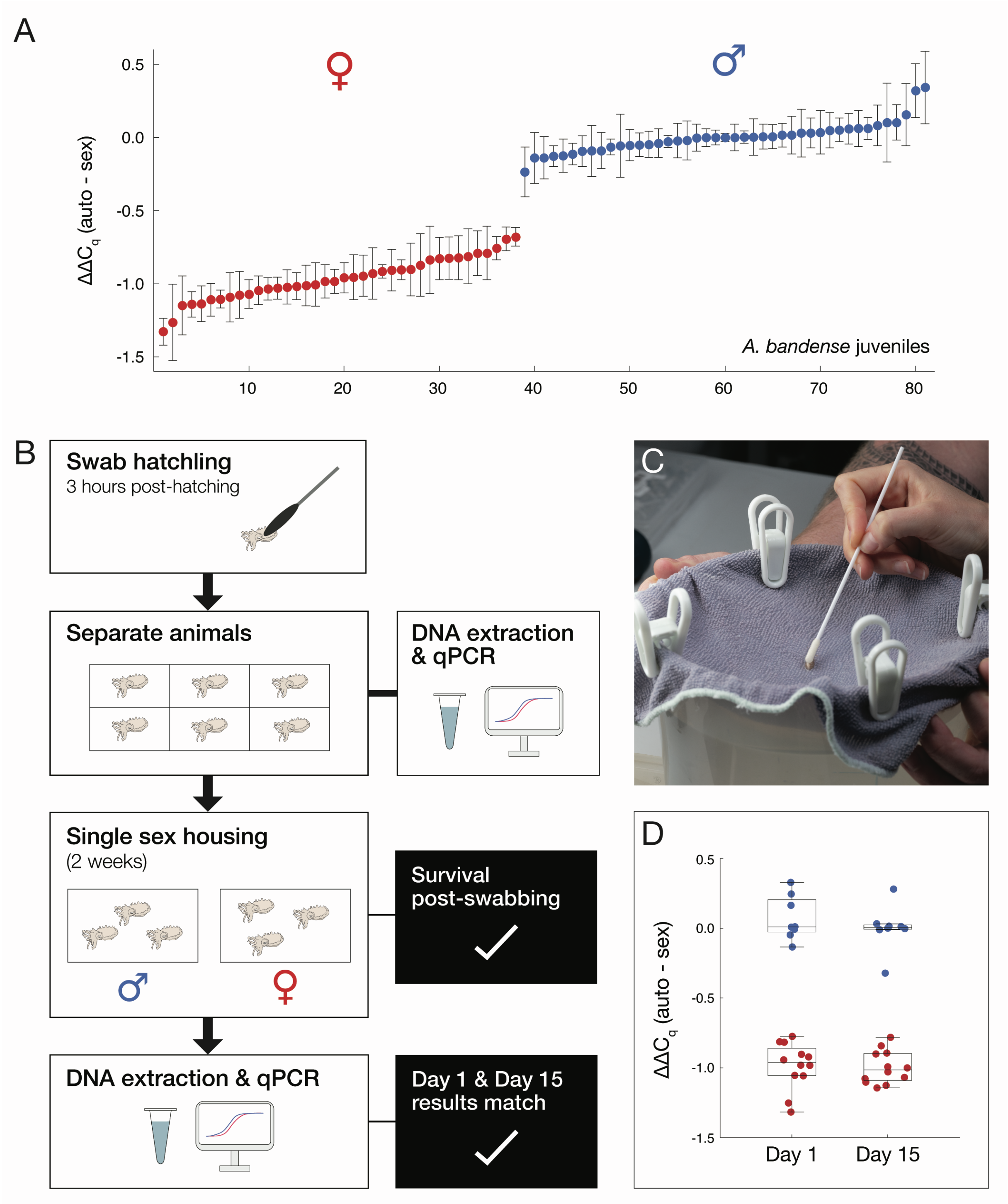

Figure S5. Quantitative PCR from non-invasive skin swabs successfully identifies sex in both adult and hatchling cuttlefish, Related to Figure 4. (A) Ranked plot of $\Delta\Delta C_q$ values comparing autosomal and sex loci in live juvenile *A. bandense* individuals, normalized such that the median value of the male cluster is set to $\Delta\Delta C_q = 0$. Each data point represents the mean ΔΔCq value of four technical replicates from a single animal (N = 81 animals), with error bars representing standard error of the mean. Predicted sex based on a $\Delta\Delta C_q$ threshold of -0.5 is shown in red (female) or blue (male); all predictions were confirmed by dissection. (B) Experimental overview: Hatchlings were swabbed within three hours post-hatching (3 hph) and separated into individual tanks. Genomic DNA was extracted from the skin swab and used for qPCR-based sex prediction. After

swabbing, the hatchlings were grouped into single-sex tanks for two weeks to monitor their survival. After two weeks, the animals were euthanized, DNA was extracted from the tissue, and used as input for qPCR to confirm that skin swabs and tissue extractions show matching sex predictions. (C) To swab hatchlings, the animals were placed on a towel soaking in a jug of seawater. (D) $\Delta\Delta C_q$ values obtained for day-1 (skin swabs) and day-15 (tissue extraction) juveniles. Each data point represents the mean ΔΔCq value of four technical replicates from a single animal (N = 20 animals). Sex predicted from day-1 samples is shown in red (female) or blue (male) and was used for single-sex housing; all animals assayed on day 15 clustered according to their day-1 prediction, using a cutoff value of $\Delta\Delta C_q$ = -0.5. Box-and-whisker plots show the median, interquartile range, and whiskers extending to the most extreme non-outlier values.
